# Long non-coding RNA LOC100129148 functions as an oncogene in human nasopharyngeal carcinoma by targeting miR-539-5p

**DOI:** 10.18632/aging.101205

**Published:** 2017-03-21

**Authors:** Kai-Yu Sun, Tao Peng, Zhe Chen, Peng Song, Xu-Hong Zhou

**Affiliations:** ^1^ Department of Otorhinolaryngology-Head and Neck Surgery, ZhongNan Hospital, Wuhan University, Wuhan 430071, Hubei, P. R. China

**Keywords:** LOC100129148, hsa-miRNA-539-5p (miR-539-5p), *KLF12*, nasopharyngeal carcinoma (NPC), tumorigenesis

## Abstract

Emerging studies have shown that long noncoding RNAs (lncRNAs) play critical roles in carcinogenesis and progression, including human nasopharyngeal carcinoma (NPC). The correlation between lncRNAs expression and NPC development has not been well identified in the recent literature. Recently, high-through put analysis reveals that LOC100129148 is highly expressed in NPC. However, whether the aberrant expression of LOC100129148 in NPC is corrected with tumorigenesis or prognosis has not been investigated. Herein, we identified that LOC100129148 was up-regulated in NPC tissues and cell lines, and higher expression of LOC100129148 resulted in a markedly poorer survival time. Over-expressed LOC100129148 favored, but silenced LOC100129148 hampered cell proliferation in NPC cells. Additionally, LOC100129148 enhanced the KLF12 expression through functioning as a competitive ‘sponge’ for miR-539-5p. Thus, our study reports a novel mechanism underlying NPC carcinogenesis, and provides a potential novel diagnosis and treatment biomarker for NPC.

## INTRODUCTION

Nasopharyngeal carcinoma (NPC), a malignancy of the epithelium that shows a variable degree of squamous differentiation where the vast majority of tumors, are undifferentiated without evidence of keratinization and are typically WHO types II and III [1,2]. Furthermore, the association with Epstein-Barr Virus (EBV) is consistent across all types of NPC although the viral presence may be difficult to demonstrate in those lesions that are WHO type [1,3,4]. Treatment for NPC patients still remains limited to combination of radiotherapy and cytotoxic agents [5]. Therefore, there is a pressing unmet need to expand the current treatment options.

Long noncoding RNAs (lncRNAs) are non-protein-coding transcripts that are > 200 nucleotides in length and reside in the nucleus or cytoplasm [6-8]. LncRNAs plays crucial roles on several systems and might be critical to various types of known cancer genes [8, 10-12]. It has been reported that lncRNAs function as important regulator in NPC. Zou et al reported that ANRIL was up-regulated in NPC and promoted the cancer progression via increasing proliferation, reprograming cell glucose metabolism and inducing side-population stem-like cancer cells [9]. LOC553103 [10], LINC01420 [11], and EWSAT1 [12] also functions as oncogenes in NPC by ceRNAs model Nevertheless, the clinical significance and biological mechanisms of lncRNAs in NPC progression are still remaining largely unknown.

LOC100129148 (NR_033999), a kind of lncRNA located in 7q34, is 463 bp in length (https://www.ncbi.nlm.nih.gov/nuccore/NR_033999). Recently, Yang and his colleagues have reported that LOC100129148 is highly expressed in NPC (4.74-fold than NP tissues) [13], while up to date, there is no related study elaborating the relevance between LOC100129148 expression and NPC progression. The role of LOC100129148 on NPC and its potential biological mechanisms still remain to be explored. Hence, we examined the expression of LOC100129148 in NPC tissues cell lines and demonstrated that LOC100129148 might play a critical role in NPC progression and prognosis as a potential prognostic biomarker.

## RESULTS

### LOC100129148 is over-expressed and associated with prognosis in NPC

**Figure 1:**
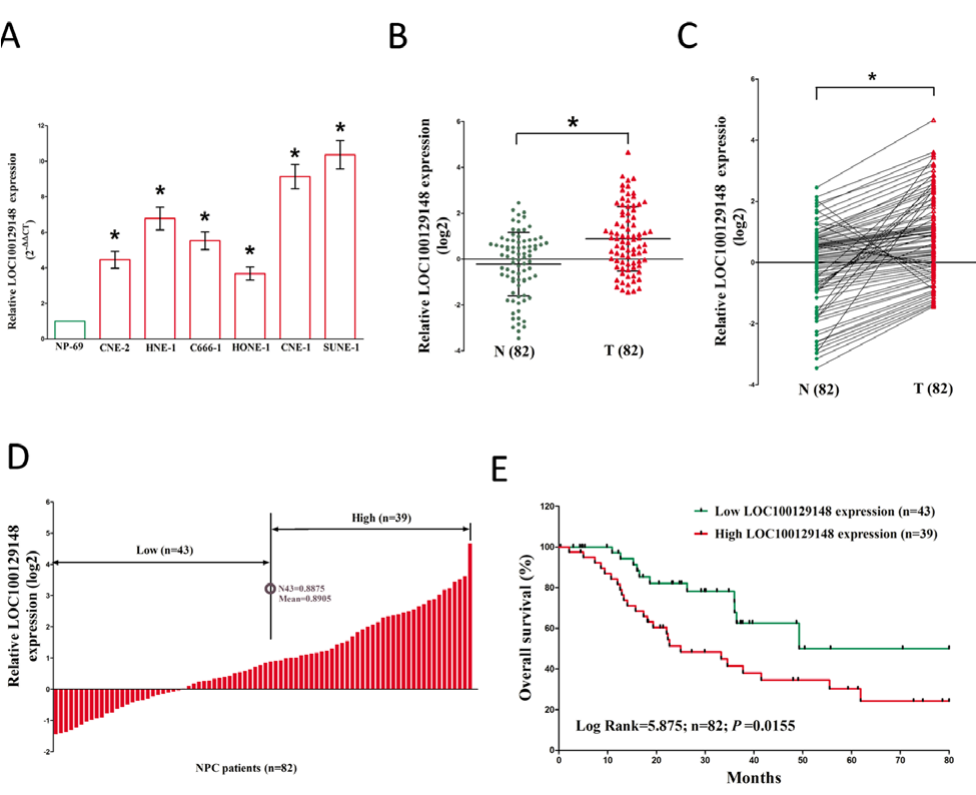
Relative LOC100129148 expression in NPC tissues and cell lines, and its clinical significance **(A)** Relative expression of LOC100129148 expression in NPC cell lines and normal NP epidermal cell. **(B)** Relative expression of LOC100129148 expression in NPC tissues (n = 82) and in paired adjacent normal tissues (n = 82). LOC100129148 expression was examined by qPCR and normalized to GAPDH expression. (shown as log2 ΔCT). **(C)** Relative expression of LOC100129148 expression in NPC tissues (n = 82) and in paired adjacent normal tissues (n = 82). LOC100129148 expression was examined by qPCR and normalized to GAPDH expression. (shown as log2ΔCT). **(D)** NPC patients were divided into a high group (≥mean, n=39) and a low group (<mean, n=43) on the basis of the cutoff value of LOC100129148 expression. **(E)** The Kaplan-Meier survival analysis indicated that LOC100129148 high expression (red line, n=39) has a worse overall survival compared to the low expression subgroup (green line, n=43). *P < 0.05. Means ± SEM are shown. Statistical analysis was conducted by student t-test.

To investigate the expression of LOC100129148 in NPC, qRT-PCR was conducted to examine LOC100129148 levels in human NPC tissues and their counterparts. Results revealed that LOC100129148 levels in 82 NPC tissues were significantly lower than that of in 82 counterparts (*P* <0.05) (Fig. 1A-B). Next, we examined LOC100129148 expression in NPC cell lines, and found that LOC100129148 was over-expressed in CNE-2, C666-1, HNE-1, CNE-1, SUNE-1, and HONE-1 cells, compared with that of in NP69 cells (a normal NP cell lines) (Fig. 1C). Among the six NPC cell lines, LOC100129148 are much higher expressed in CNE-1 and SUNE-1 cells, thus, CNE-1 and SUNE-1 cells were chose to conduct the following experiments. Then, NPC patients were divided into a high group (≥mean, n=39) and a low group (<mean, n=43) on the basis of the cutoff value of LOC100129148 expression (Fig. 1D). Moreover, Kaplan-Meier analysis indicated that high LOC100129148 expression was related to a poorer OS (log-rank test, *P* =0.0155, Fig. 1E). These results demonstrated that high LOC100129148 expression was related to poor prognosis, and over-expression of LOC100129148 might be essential in NPC progression.

### LOC100129148 promotes growth of NPC cells *in vitro* and *in vivo*

**Figure 2:**
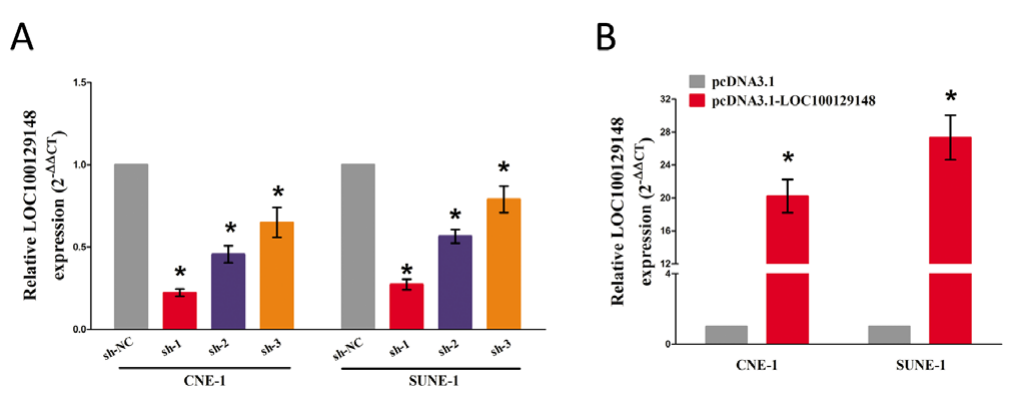
**(A-B)** Relative LOC100129148 expression after transfection with sh-LOC100129148 or pcDNA3.1-LOC100129148.

We also studied the impact of LOC100129148 on the proliferation of NPC cells. To this end, CNE-1 and SUNE-1 cells were transfected with control shRNA or shRNAs against LOC100129148, i.e., sh-LOC100129148#1 (sh-1), sh-LOC100129148#2 (sh-2), and sh-LOC100129148#3 (sh-3), pcDNA3.1 control and pcDNA3.1-LOC100129148. The qRT-PCR results indicated that sh-1, sh-2, and sh-3 effectively knocked down LOC100129148, and pcDNA3.1-LOC100129148 significantly increased LOC100129148 expression (Fig. 2A-B).

**Figure 3:**
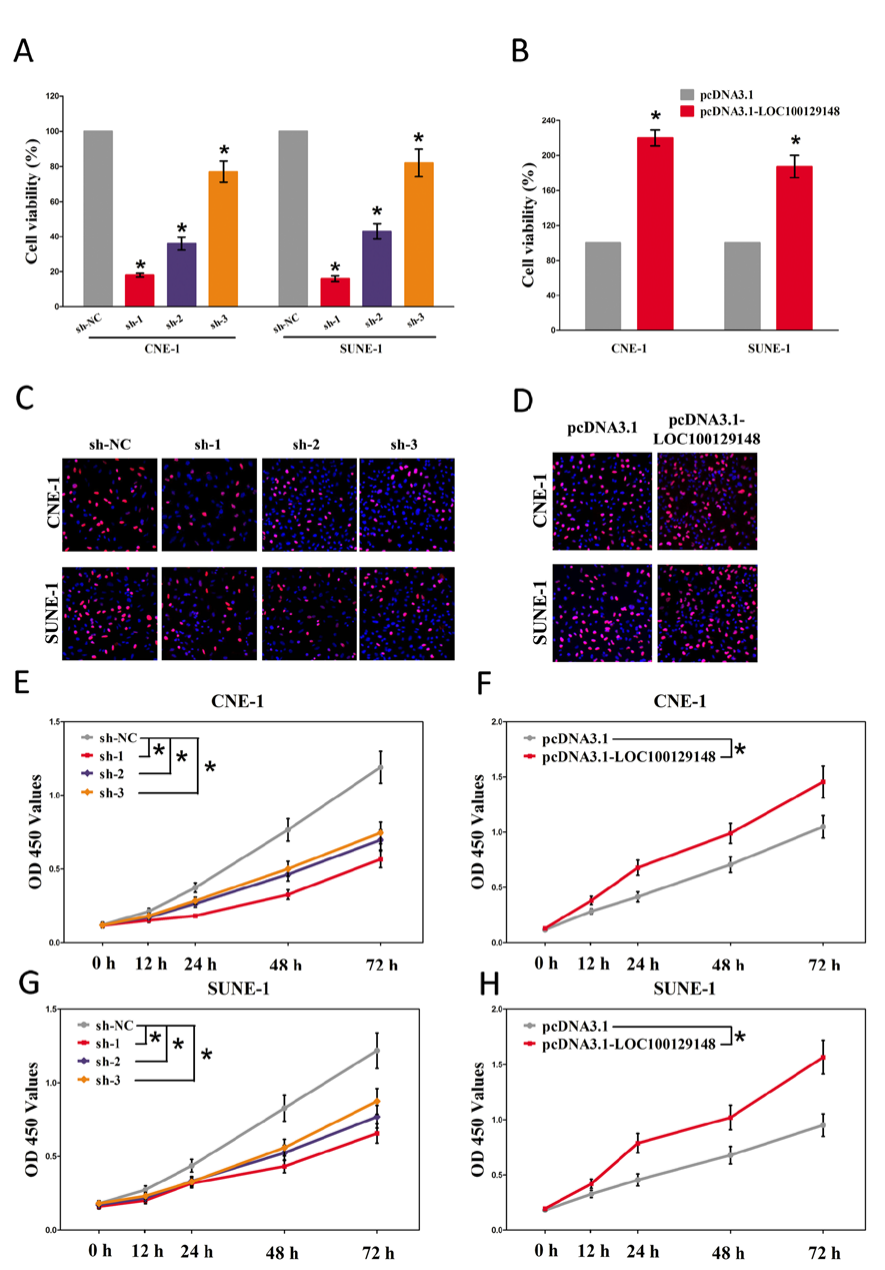
LOC100129148 promotes tumor NPC cell growth *in vitro* **(A-B)** Statistical analysis of trypan blue staining. **(C-D)** Shown is representative photomicrograph of BrdU staining assay after transfection for fourteen days. **(E-H)** CCK8 assays of CNE-1 and SUNE-1 cells after transfection. Assays were performed in triplicate. *P < 0.05. Means ± SEM are shown. Statistical analysis was conducted using student t-test.

And then, trypan blue staining, BrdU staining as well as CCK8 assay were conducted to explore the role of LOC100129148 on NPC cell growth, and results demonstrated silence of LOC100129148 induced a reduction in the cell growth of CNE-1 and SUNE-1 cells than that of in their blank counterparts (Fig. 3C, E, and G). However, overexpression of LOC100129148 exhibited a significant increase in the cell growth of CNE-1 and SUNE-1 cells than their blank counterparts (Fig. 3D, F, and H).These results clearly demonstrate that LOC100129148 significantly facilitates cell growth in NPC cells.

**Figure 4:**
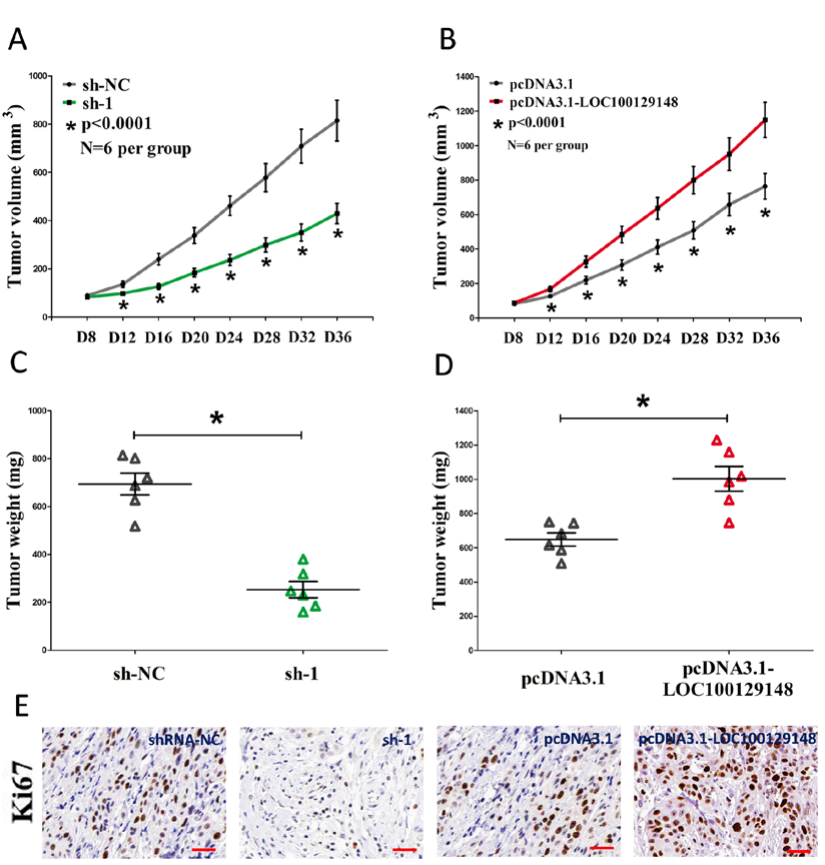
LOC100129148 promotes tumor NPC cell growth in vivo **(A-B)** Tumor volume subcutaneous implantation models of CNE-1 cell are shown. **(C-D)** Tumor weight subcutaneous implantation models of CNE-1 cell are shown. **(E)** Immunohistochemistry of Ki67 in tumors isolated from shRNA-NC, sh-1, pcDNA3.1, and pcDNA3.1-LOC100129148 groups. Assays were performed in triplicate. *P < 0.05. Means ± SEM are shown. Statistical analysis was conducted using student t-test.

To further investigate whether knockdown of LOC100129148 expression could affect tumor growth in vivo, CNE-1 cells stably transfected with sh-1 or shRNA-NC, pcDNA3.1-LOC100129148 or pcDNA3.1-empty vectors were injected into male nude mice. Thirty-six days after inoculation, the tumor size and weight in the sh-1 group were markedly smaller/lighter in comparison to that of in their counterparts group, and the tumor size and weight in the pcDNA3.1-LOC100129148 group was markedly larger/heavier than that of in pcDNA3.1-empty vectors group (Fig. 4A-D). To further investigate the in vivo effects of LOC100129148, tumor cell proliferation was assessed using proliferation-related nuclear antigen Ki67 immunoreactivity assay. We found that up-regulation of LOC100129148 promoted tumor cell proliferation, and down-regulation of LOC100129148 inhibited tumor cell proliferation (Fig. 4E). Taken together, these results demonstrate that LOC100129148 plays a crucial role on NPC progression.

### LOC100129148 functions as a ceRNA of miR-539-5p in NPC

Increasing of publications reported lncRNA might function as a ceRNA or a molecular sponge in regulating the biological functions of miRNA. To find miRNAs interacted with LOC100129148, we analyzed the results of miRDB (http://mirdb.org/cgi-bin/custom_predict/customDetail.cgi) to predict potential miRNAs (results were shown in Table 1. In miRDB, miRNAs with target score≥50 were selected.

**Table 1:** Predicted results using miRDB (target score≥50)

Target Rank	Target Score	miRNA Name	Gene Symbol
1	92	hsa-miR-539-5p	submission
2	90	hsa-miR-1252-3p	submission
3	77	hsa-miR-6510-5p	submission
4	75	hsa-miR-1227-5p	submission
5	65	hsa-miR-6845-5p	submission
6	65	hsa-miR-6762-5p	submission
7	63	hsa-miR-4640-5p	submission
8	60	hsa-miR-6823-5p	submission
9	55	hsa-miR-4650-3p	submission
10	50	hsa-miR-6771-5p	submission

**Figure 5:**
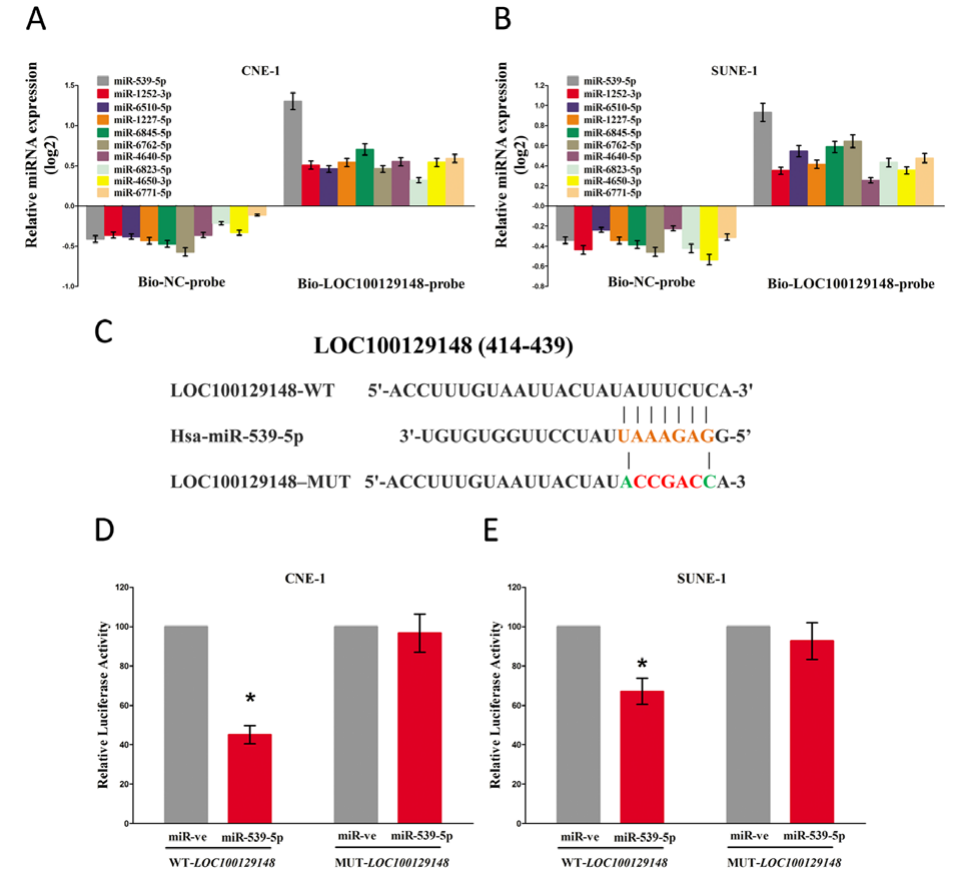
LOC100129148 is a direct target of miR-539-5p **(A-B)** Detection of targeted miRNAs using qRT-PCR in the sample pulled down by biotinylated FTH1P3 probe. **(C)** Sequence alignment of miR-539-5p with the putative binding sites within the wild-type regions of LOC100129148. **(D-E)** The luciferase report assay demonstrated that overexpression of miR-539-5p could reduce the intensity of fluorescence in CNE-1 and SUNE-1 cells transfected with the LOC100129148-WT vector, while had no effect on the LOC100129148-MUT vector. Assays were performed in triplicate. *P< 0.05. Means ± SEM are shown. Statistical analysis was conducted using student t-test.

To identify which miRNA was the most one that enrichment in LOC100129148, we conducted a pull-down assay using a biotin-labeled specific LOC100129148 probe. And a biotin- labeled NC probe was used as a negative control. Then, qRT-PCR was conducted after precipitate. Results revealed that miR-539-5p was much richer in precipitate of LOC100129148 probe than that of in NC probe, and also much richer than that of in other predicted miRNAs (including miR-1252-3p, miR-6510-5p, miR-1227-5p, miR-6845-5p, miR-6762-5p, miR-4640-5p, miR-6823-5p, miR-4650-3p, and miR-6771-5p) (Fig. 5A-B). Moreover, we also conducted dual-luciferase reporter assay to further identify whether LOC100129148 was a functional target for miR-539-5p. Our data demonstrated miR-539-5p may suppress the luciferase activity of pmirGLO-LOC100129148-WT, but it has not affected the luciferase activity of pmirGLO-LOC100129148-MUT (Fig. 5C-E), which implied that miR-539-5p directly bound to LOC100129148 at the recognized sites.

**Figure 6:**
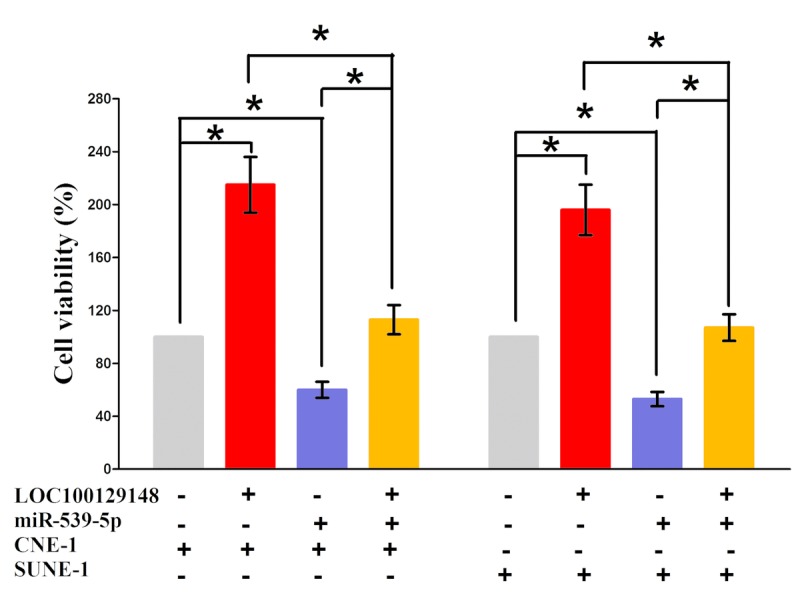
Up-regulated miR-539-5p in CNE-1 and SUNE-1 cells, which stably over-expressed LOC100129148, largely reversed the favorable effects of LOC100129148 on cell proliferation Assays were performed in triplicate. *P< 0.05. Means ± SEM are shown. Statistical analysis was conducted using student t-test.

In addition, we also conducted trypan blue staining assay to explore the interaction between miR-539-5p and LOC100129148 on NPC cell growth, and results demonstrated miR-539-5p suppressed cell growth both in CNE-1 and SUNE-1 cells, while when co-transfection of miR-539-5p and pcDNA3.1-LOC100129148, the growth-inhibitory role of miR-539-5p was reversed, but the growth expedited role of LOC100129148 was also hampered (Fig. 6). These data demonstrated that LOC100129148 facilitated cell growth via functioning as a ceRNA for miR-539-5p in NPC cell lines.

### LOC100129148's oncogenic roles are partially via sponging miR-539-5p, and then activating KLF12

**Figure 7:**
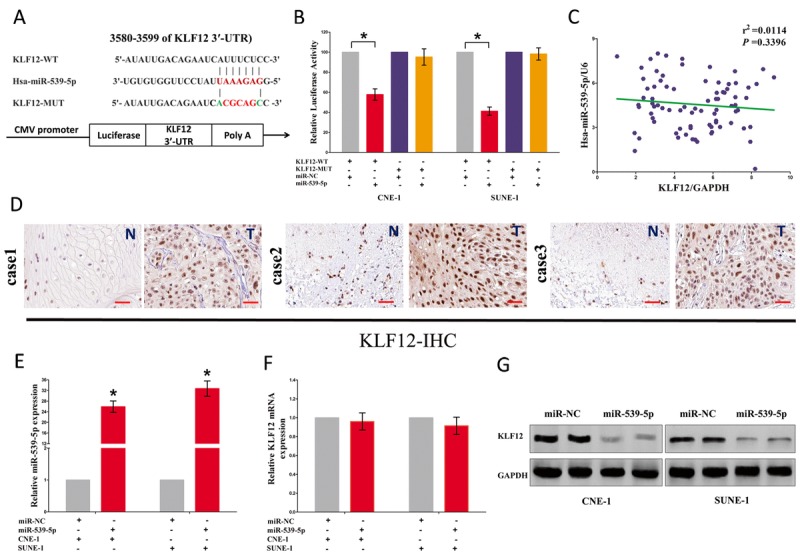
LOC100129148's oncogenic activity is in part through negative regulation of miR-539-5p, and then activation of KLF12 in NPC cells **(A)** The 3'-UTR of KLF12 harbors one miR-539-5p cognate site. **(B)** Relative luciferase activity of reporter plasmids carrying wild-type or mutant KLF12 3^'^-UTR in CNE-1 and SUNE-1 cells co-transfected with negative control (NC) or miR-539-5p mimic. **(C)** mRNA expression of KLF12 and miR-539-5p in NPC tissues. **(D)** KLF12 is highly expressed in NPC tissues (T) that NP tissues (N). **(E)** Relative miR-539-5p expression after transfection with miR-NC and miR-539-5p. **(F)** Relative KLF12 mRNA expression after transfection with miR-NC and miR-539-5p. **(G)** Relative KLF12 protein expression after transfection with miR-NC and miR-539-5p. Assays were performed in triplicate. *P < 0.05. Means ± SEM are shown. Statistical analysis was conducted using student One-Way ANOVA test.

To explore the function of miR-539-5p on NPC, we screen Targetscan, miRanda, PicTar to select potential predicted targets of miR-539-5p. We identified the top 100 potential targets, and among these genes, we found a well-known oncogene, KLF12, which was up-regulated in a large number of malignancies. These revealed that KLF12 could be a direct target of miR-539-5p in NPC (Fig. 7A). Next, we used luciferase reporter assays to verify whether KLF12 expression are really regulated by miR-539-5p, and results demonstrate that miR-539-5p inhibits luciferase activity in CNE-1 cells and SUNE-1 cells at the reporter plasmid with a WT KLF12 3'-UTR, but no significant inhibition was observed at the reporter plasmid with a mutant KLF12 3'-UTR (Fig. 7B). However, our results also demonstrated the mRNA of KLF12 expression had no significant correction with the expression of miR-539-5p in NPC samples (r2 =0.0144, *P* =0.3396) (Fig. 7C). We also discovered that KLF12 was highly expressed in NPC tissues than that of in normal NP tissues, as demonstrated by IHC assay (Fig. 7D). We also found that transfection with miR-539-5p significantly up-regulated the miR-539-5p expression in CNE-1 and SUNE-1 cells (Fig. 7E). Moreover, miR-539-5p decreased the protein expression but had no influence on the mRNA expression for KLF12 in CNE-1 and SUNE-1 cells (Fig. 7F-G).

**Figure 8:**
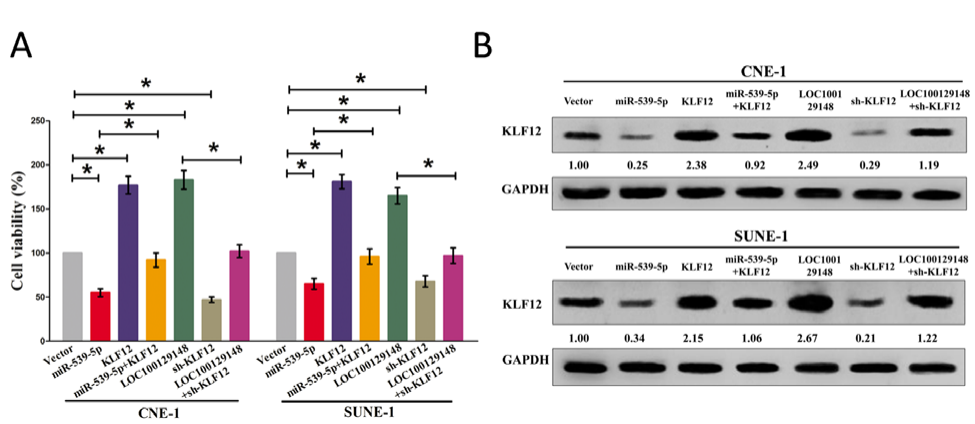
LOC100129148's oncogenic activity is in part through negative regulation of miR-539-5p, and then activation of KLF12 in NPC cells **(A)** Statistical analysis of trypan blue staining. **(B)** Protein expression of KLF12 in miR-539-5p, sh-KLF12, LOC100129148, or LOC100129148+sh-KLF12 treated CNE-1 and SUNE-1 cells. Assays were performed in triplicate. *P < 0.05. Means ± SEM are shown. Statistical analysis was conducted using student One-Way ANOVA test.

We then explored the role and mechanism of miR-539-5p on NPC cell growth. Results of Trypan blue staining revealed miR-5395p treatment suppressed cell growth, and pcDNA3.1-KLF12 treatment facilitated cell growth in CNE-1 and SUNE-1 cells (Fig. 8A). However, when treated CNE-1 and SUNE-1 cells with miR-539-5p plus pcDNA3.1-KLF12, the advantageous role of KLF12 on cell growth was reversed by miR-539-5p, and the growth-inhibitory effect of miR-539-5p was inversed by KLF12 over-expression (Fig. 8A). These data demonstrated that miR-539-5p suppressed cell growth by directly targeting 3'-UTR of KLF12 mRNA. Additionally, pcDNA3.1-LOC100129148 treatment reversed the growth inhibitory effect of sh-KLF12 in CNE-1 and SUNE-1 cells (Fig. 8A), which demonstrated that LOC100129148 facilitated cell growth partially through up regulation of KLF12. Furthermore, we next explored the role of LOC100129148 and miR-539-5p on the protein expression of KLF12. Results demonstrated that both miR-539-5p and sh-KLF12 treatment suppressed protein expression of KLF12, while both pcDNA3.1-LOC100129148 and pcDNA3.1-KLF12 treatment significantly enhanced protein expression of KLF12 in CNE-1 and SUNE-1 cells (Fig. 8B), respectively. However, when treated CNE-1 and SUNE-1 cells with pcDNA3.1-LOC100129148 plus sh-KLF12, the beneficial role of LOC100129148 on protein expression of KLF12 was suppressed by knockdown of KLF12, and the negative effect of sh-KLF12 was alleviated by over-expression of LOC100129148 (Fig. 8B). These findings suggest that the oncogenic role of LOC100129148 is mediated by miR-539-5p-KLF12 axis in NPC.

## DISCUSSION

LncRNAs participate in many biological processes and many studies have implicated that abnormal expression of lncRNAs is closely related to the occurrence and development of malignant tumors [6-8, 10-12, 14-17]. Yang and his colleagues have reported that LOC100129148 is highly expressed in NPC (4.74-fold than NP tissues) [18]. However, the roles and mechanisms of LOC100129148 in NPC have not been well elaborated. Our present study added new evidence that over-expression of LOC100129148 owned oncogenic roles in NPC. LOC100129148 was revealed as a direct target of miR-539-5p, and there was an interactive suppression between them. LOC100129148's function as an oncogene to facilitate tumor progression was partially attributed to its ability to acting as a ceRNA for miR-539-5p, and subsequent to activating of the KLF12 signaling pathway in NPC. Thus, our study contributes to an increasing of literatures supporting the importance of non-annotated lncRNA species in the field of cancer research.

In this study, we reported a novel functional lncRNA LOC100129148 which was significantly high expressed in NPC samples and correlated with a poor prognosis of NPC patients. Our report is the first one to directly explore the association between LOC100129148 expression and NPC. Herein, we found LOC100129148 expression in NPC tissues was significantly higher than that of in NP tissues. Our study also revealed a correction between LOC100129148 levels and NPC prognosis or therapeutic outcome. A strong correction of high LOC100129148 expression in tumors with poor survival was confirmed in 82 NPC samples, revealing that LOC100129148 expression levels could be as a useful prognostic biomarker to help identify patients who are at a higher risk of NPC progression. In addition, LOC100129148 overexpression significantly increased NPC cell viability and growth in vitro, while LOC100129148 knockdown reversed it. In conclusion, our data indicate that LOC100129148 may function as an oncogene and play a critical effect in NPC development and progression.

Although LOC100129148 has been suggested to act as an oncogene, the underlying mechanism by which LOC100129148-mediated gene expression participates in tumorigenesis remains to be clarified. In our present study, we discover the underlying molecular mechanism of LOC100129148 on NPC progression, by functioning as “molecular sponges” to regulate microRNAs. A handle of lncRNAs have been evaluated to play a crucial effect in multiple processes in cells through acting as ceRNAs to regulate microRNAs, including RMRP, NEAT1 [19], and RSU1P2 [20] and so on. MiRNAs play crucial roles in cancers [18, 21-37]. In our study, we investigated the effect of LOC100129148 in NPC cell lines and discovered that LOC100129148 involved in the ceRNA regulatory network and functioned as endogenous miRNA sponges to bind to miR-539-5p and regulated its function. Recent studies indicated miR-539-5p showed tumor suppressive role on osteosarcoma [38], prostate cancer [39], and thyroid cancer [40], while their role on NPC had not been investigated. In our present study, miR-539-5p was decreased expression in NPC tissues and cell lines, and miR-539-5p inhibited growth in NPC cell lines. Moreover, biotin-avidin pull-down system demonstrated LOC100129148 could pull down miR-539-5p. In addition, our study also revealed that miR-539-5p could reverse the favorable roles of LOC100129148 on cell growth in NPC cell lines, which demonstrated LOC100129148 played its favorable role on NPC progression, at least in part, through inhibiting miR-539-5p.

Having shown the critical role of miR-539-5p on suppressing NPC progression, we searched for the potential gene effectors involved in its function. MiR-539-5p can regulate numerous of target genes. Recent study indicated that miR-539 inhibits thyroid cancer cell migration and invasion by directly targeting CARMA1 [40], induces cell cycle arrest in nasopharyngeal carcinoma by targeting cyclin-dependent kinase 4 [41]. MiR-539 inhibits prostate cancer progression by directly targeting SPAG5 [39]. But among all of the predicted target genes for miR-539-5p, we found that KLF12 acted as a crucial effector of miR-539-5p. Aberrant KLF12 expression has been associated to several types of cancers [42, 43]. In our study, highly expression of KLF12 expression was found in NPC than pair-matched adjacent NP tissues. Using bioinformatics, we verified KLF12 as a direct target of miR-539-5p, and luciferase reporter assays confirmed that miR-539-5p targeted KLF12 mRNA at its 3'-UTR. Moreover, our results also demonstrated miR-539-5p exerted its tumor suppressive role on NPC through targeting KLF12. And LOC100129148 facilitated NPC cell growth through up-regulated the expression of KLF12.

In summary, the findings presented in this study suggested that LOC100129148 expression was commonly high expressed in NPC. Furthermore, high expression of LOC100129148 was an independent poor prognostic for NPC patients. High-expressed LOC100129148 is an oncogenic lncRNA that facilitates the oncogenesis and progression of NPC through miR-539-5p-KLF12 axis. The present results elucidate an underlying mechanism of the oncogenic role for LOC100129148 in NPC, indicating that further investigation of LOC100129148 might lead to the development of novel tumor therapies in NPC.

## MATERIALS AND METHODS

### Ethical statement

For the analyzed tissue specimens, all patients gave informed consent to use excess pathological specimens for research purposes. The protocols employed in this Subjects Committee. The use of human tissues was approved by the institutional review board of the Wuhan University and conformed to the Helsinki Declaration and to the local legislation. Patients offering samples for the study signed informed consent forms.

### Tissue collection

82 cases of fresh NPC tissues and 82 non-cancerous nasopharyngitis (NP) tissues were snap-frozen and stored in liquid nitrogen until further use for qRT-PCR assay. Elective surgery was carried out on these patients at ZhongNan Hospital of Wuhan University (Wuhan, China). The use of tissues for this study has been approved by the ethics committee of ZhongNan Hospital of Wuhan University. Before using these clinical materials for research purposes, all the patients signed the informed consent. None of these patients received any pre-operative chemotherapy or radiotherapy.

### Cell Culture and transfection

The human NPC cell lines, namely, SUNE-1, CNE-1, HNE-1, CNE-2, C666-1 and HONE-1 were cultured in RPMI-1640 (Invitrogen, Carlsbad, CA, USA) supplemented with 10%. fetal bovine serum (FBS). The human immortalized nasopharyngeal epithelial cell line NP69 was cultured in keratinocyte/serum-free medium (Invitrogen) supplemented with bovine pituitary extract. LOC100129148, sh-LOC100129148, miR-539-5p, or sh-KLF12, were purchased from GenePharma Co.,Ltd. (Shanghai, China). Complete medium without antibiotics was used to culture the cells at least twenty-four hours prior to transfection. The cells were washed with 1× PBS (pH7.4) and then transiently transfected with 100 nM NC or LOC100129148, sh-LOC100129148, miR-539-5p, or sh-KLF12, using Lipofectamine™ 2000 (Invitrogen, Carlsbad, CA, USA) according to the manufacturer's instructions.

### Western blot

The protein in cells and tissues were extracted using RIPA Lysis Buffer and were separated using SDS-PAGE. After transferred to a PVDF membranes, the membranes were blocked with 5% non-fat milk and incubated with the primary antibodies (Abcam, UK) overnight at 4℃. Then the bands were probed with secondary antibody (Icllab, USA) and visualized by chemiluminescence (Millipore, MA, USA). Rabbit polyclonal antibody against human KLF12 and GAPDH were purchased from Abcam.

### EdU assay

For cell proliferation assays, the cells were incubated with 5-ethynyl-20-deoxyuridine (EdU) (Invitrogen, USA) for 5 h, followed by incubation with 300 μl of 1×Apollo® reaction cocktail for 30 min. Then, the DNA contents of the cells were stained with Hoechst 33342 for 30 min and visualized under a fluorescence microscope.

### qRT-PCR

Total RNA was extracted with TRIzol reagent in accordance with the manufacturer's instructions (Invitrogen, CA, USA). cDNA was synthesised with the PrimeScript RT reagent Kit (Promega, Madison, WI, USA). Real-time PCR was carried out in a total volume of 10 μl, including 8 μl of TaqMan Power SYBR Green PCR Mix (Invitrogen), 0.5 μl of each primer at 25 μM, and 1 μl of cDNA. The quantitative RT-PCR was carried out on the Roche LightCycler® 96 (LC96) real-time PCR platform using the 2^-∆∆CT^ method. Gene expression results were normalized by internal control GAPDH. Each sample was tested in triplicate.

### Immunohistochemistry

Immunohistochemistry of patient tissue sections was performed as described recently [44-46]. The dewaxed 5-μm sections were subjected to an antigen-demasking procedure of brief, high temperature heating of the sections immersed in citrate buffer. Endogenous peroxidases were blocked with 0.3% hydrogen peroxide, and nonspecific binding was blocked with 5% normal goat serum and 2% BSA in phosphate-buffered saline (PBS). Sections were then incubated for 2 hours at room temperature with anti- KLF12 or Ki67 antibody (1:50; Abcam). After washing with PBS, sections were incubated with biotinylated secondary antibody, followed by a further incubation with the streptavidin–horseradish peroxidase complex. The sections were then immersed in DAB for5 to 10 minutes, counterstained with 10% Mayer hematoxylin, dehydrated, and mounted in crystal mount.

### Dual luciferase assay

The putative miR-539-5p target binding sequence in LOC100129148 (KLF12) and its mutant of the binding sites were synthesized and cloned into the downstream of the luciferase gene to generate the wild-type (wt) reporter plasmid and mutated-type (Mut) reporter plasmid. 48 h after transfection, the luciferase activity was measured using the Dual-Luciferase Reporter Assay System (Promega) according to the manufacturer's instructions. The relative luciferase activity was normalized to renilla luciferase activity.

### CCK8 Assay

CCK8 Assay was carried out using the protocol described previously [7, 18, 21, 47]. Briefly, cell growth was measured using the cell proliferation reagent WST-8 (Roche Biochemicals, Mannheim, Germany). After plating cells in 96-well microtiter plates (Corning Costar, Corning, NY) at 1.0× 10^3^ /well, 10 μL of CCK8 was added to each well at the time of harvest, according to the manufacturer's instructions. One hour after adding CCK8, cellular viability was determined by measuring the absorbance of the converted dye at 450 nm.

### RNA immunoprecipitation assay

Magna RIP™ RNA-Binding Protein Immunoprecipitation Kit (Millipore, USA) was applied to perform the RIP assay according to the manufacturer's instructions. Whole-cell lysate was harvested and subsequently incubated with RIP buffer containing magnetic beads conjugated with anti-Ago2 antibody (Abcam, UK) or negative control IgG (Sigma-Aldrich, USA). After incubated with Proteinase K, the immunoprecipitated RNA was isolated. The RNA associated with Ago2 antibody was extracted and analyzed by qRT-PCR.

### Statistical analysis

All experiments were repeated for three times independently. Results were shown as the means ± standard error mean (SEM). Two independent sample t-test or One-Way Analysis of Variance (ANOVA) was performed using SPSS 20.0 software to assess significant differences in measured variables among groups. A value of *P* <0.05 was considered to indicate a statistically significant difference.
